# Cross-Cultural Adaptation, Validity, and Reliability of the Patient-Rated Michigan Hand Outcomes Questionnaire for Thai Patients

**DOI:** 10.1155/2018/8319875

**Published:** 2018-11-18

**Authors:** Jananya P. Dhippayom, Piyawat Trevittaya, Andy S. K. Cheng

**Affiliations:** ^1^Department of Occupational Therapy, Faculty of Associated Medical Sciences, Chiang Mai University, Chiangmai 50200, Thailand; ^2^Department of Rehabilitation Sciences, Faculty of Health and Social Sciences, Hong Kong Polytechnic University, Hung Hom, Kowloon, Hong Kong

## Abstract

**Introduction:**

The Michigan Hand Outcomes Questionnaire (MHQ) is a patient-rated hand outcome instrument. It is widely used in orthopedic and neurological conditions of the hands and upper limbs. To gain more knowledge on hand outcomes from a Thai patient perspective, an MHQ-Thai version is required.

**Purpose of the Study:**

The study is aimed at translating and cross-culturally adapting the MHQ into Thai and at examining the validity and reliability of the translated version.

**Methods:**

The Beaton protocol for cross-cultural adaptation of self-reported measures was used in the translation process. Three occupational therapists were asked to assess content validity while 30 participants were asked to fill in the questionnaire in order to assess construct validity, internal consistency, and test-retest reliability.

**Results:**

All six domains of the MHQ were translated into Thai without any major problems. However, items related to the characteristics of the patients were adapted to suit the Thai context. The MHQ-Thai version had good content validity (IOC 0.972). The construct validity revealed a low-to-high correlation between every subscale of the MHQ-Thai version. The intraclass correlation coefficient (ICC) of the test-retest reliability for the six domains ranged from 0.788 to 0.956, with excellent correlation (ICC = 0.953) for the total score. Cronbach's alpha was 0.835 for the total score of the MHQ-Thai version, indicating good internal consistency.

**Discussion and Conclusions:**

MHQ was successfully cross-culturally adapted into Thai. The MHQ-Thai version is a valid and reliable instrument for evaluating the self-perception of Thai people who have hand and upper limb injuries.

## 1. Introduction

Hands are complex limbs that consist of bones, nerves, muscles, tendons, ligaments, arteries, and veins [1]. An individual requires sound structure and function in the hands and upper limbs to perform activities in daily life. Any impairment, such as neurological or musculoskeletal injury, to the hands and upper limbs could have an impact on their functioning. Hand injuries also have an impact on a patient's life, including activities of daily living, work, and leisure. The ability to function effectively and independently has long been a focus for occupational therapy [2]. Occupational therapists need to deal with people who have hand injuries to enable them to regain maximum use and capability [3]. Consequently, outcome measures have become necessary and important tools, in assessing the efficacy of hand rehabilitation programs [3]. Two main kinds of outcome measures have been proposed, namely, performance-based outcome measures and self-reported outcome measures [3]. The performance-based outcome measures, such as the Jebsen Hand Function Test and the Minnesota Manual Dexterity Test, are tools that require patients to perform a set of functional tasks. In contrast, the self-reported outcome measures are those that require the patient to complete questionnaires, rating his or her overall performance on a predetermined set of functional tasks [3]. Some examples of self-reported outcome measures used in hand rehabilitation are the Canadian Occupational Performance Measure (COPM), the Disabilities of Arm, Shoulder and Hand (DASH) questionnaire, and the health-related quality of life-Short Form 36 (SF 36) [3]. Even though performance-based outcome measures convey more accurate representations of a patient's actual ability, the information given by patients themselves is also important: how they feel and how they think about their hands after having injuries. By adding the subjective information from patients on the actual performance, occupational therapists can have a more holistic picture of patients with hand injuries. The Michigan Hand Outcomes Questionnaire (MHQ) is a self-reporting outcome measure that is widely used in assessing hand injuries [4]. It was developed in 1998 and has become a useful tool to measure health status domains, including overall hand function, activities of daily living (ADL), pain, work performance, aesthetics, and satisfaction [4]. It comprises 37 items with five rating scales from 1 to 5. Each domain is scored and converted to values from 0 to 100 [4]. A higher numerical score means better results, except for the pain domain in which a higher score represents more pain. It can measure impairment of each side of the hand separately [5]. The validity and responsiveness of the MHQ have been proven for a variety of hand conditions [4]. Also, it has been translated into several languages such as Chinese, Dutch, German, and Japanese [6]. At present, it has not yet been translated into Thai. Therefore, to gain more knowledge from Thai patients with hand injuries, this study is aimed at translating the MHQ into Thai and investigating the validity and reliability of the MHQ-Thai version.

## 2. Materials and Methods

The study was approved by the ethical committee of the Faculty of Associated Medical Sciences, Chiang Mai University, Thailand.

### 2.1. Translation

The permission to translate the MHQ was given by the developer, the Department of Plastic Surgery, University of Michigan School of Medicine. The study followed the Beaton protocol for the cross-cultural adaptation of self-reporting measures [7]. The MHQ was translated following the six steps developed by Beaton:

**Step 1 .**

Initial translation. The questionnaire was translated from English to Thai by bilingual translators whose mother tongue was Thai [7]. In the study, two translators from different backgrounds (one was a researcher and the other was from a nonmedical background) independently translated the MHQ into Thai. The outcomes of Step 1 were the MHQ-T1 and the MHQ-T2.

**Step 2 .**

Merge translation. The two translations were merged into a single translation. Then, the differences between the two translations were discussed and addressed by the authors. The merged version was called the MHQ-T12.

**Step 3 .**

Back translation. The merged translation (MHQ-T12) was translated back into English. Then, without being given any information on the original version, two native English speakers were asked to produce two independent English versions (MHQ-BT1 and MHQ-BT2). This step was to ensure that the translated version reflected the same item content as the original version.

**Step 4 .**

Expert committee. A panel of experts, including an occupational therapist, a language professional, and four translators, discussed MHQ-BT1 and MHQ-BT2 in order to produce a prefinal version of the questionnaire for field testing. The aim of the discussion was to achieve semantic, idiomatic, experiential, and conceptual equivalence.

**Step 5 .**

Testing of the prefinal version. Before field testing, content validity of the prefinal version was evaluated. Three occupational therapists, who had been working with patients with hand injuries for more than five years, were asked to score each item for the degree to which it measured the specific objective listed by the test developer. The scoring system for each questionnaire item was as follows:

- +1 = clearly measuring
- 0 = unclear
−1 = clearly not measuring

The item-objective congruence (IOC) value for each item was calculated using the summation of scores from each occupational therapist, divided by the number of occupational therapists.

For further confirmation, the prefinal version was tested on ten patients who had hand injuries due to orthopedic conditions. They were asked to rate how fully they could understand the items and how easily they could answer the questionnaire.

**Step 6 .**

Submission of documentation to the developers. In the last step, all the reports and forms were submitted to the developers to allow them to keep track of the translated version.

### 2.2. Psychometric Testing of the MHQ-Thai Version

The content and construct validity, internal consistency, and test-retest reliability were examined. The number of participants of 30 was the minimum sample size suggested by many statisticians [8]. Accordingly, 30 patients with hand injuries were recruited to the study. They were all outpatients from occupational therapy units of four hospitals in three provinces, including Chiangmai, Phitsanulok, and Bangkok. Using the purposive sampling method, they were invited to participate in the study if they met the inclusion criteria of having a hand injury from an orthopedic condition, being aged 18 years or above, and being able to read and speak Thai. They were asked to complete the MHQ-Thai version twice, with a one- to two-week interval in between.

## 3. Data Analysis

The gathered data were analyzed using SPSS for Windows version 17. The patients' characteristics, MHQ scores, and distribution of the scores were calculated. The content validity was assessed using the item-objective congruence (IOC) value. An IOC value of 0.5 or more was considered satisfactory [9]. To test the construct validity of the MHQ-Thai version, the responses of each domain were tested against the other domains in the MHQ to determine if each scale behaved in the expected manner by using Spearman's correlation coefficients. Interpretation of the size of a correlation coefficient was determined by following the procedures set out by Hinkle et al. [10]. Cronbach's alpha coefficient was used to assess internal consistency. An acceptable internal consistency, defined by Cronbach's alpha coefficient, ranged from 0.7 to 0.95 [11]. The test-retest reliability was calculated using the intraclass correlation coefficient (ICC) (3,1) [12]. The ICC interpretation, as described by Koo [12], was used to indicate the level of reliability.

## 4. Results

### 4.1. Translation and Cross-Cultural Adaptation Process

Even though both of the forward translation versions (MHQ-T1 and MHQ-T2) differed in style with regard to sentence composition, the meaning was found to be the same. After discussion, the MHQ-T12 was finalized. Then, back translation was carried out and two back translated versions were produced. Both were confirmed to have retained the questionnaire's original meaning. Turning to the process undertaken by the expert committee, the meaning of the word “job” in Thai, in the section on demographic data, was discussed, and one word was selected that fitted best with the original questions.

#### 4.1.1. Content Validity

An average IOC of 0.972 indicated good content validity [8]. The IOC values of each item ranged from 0.67 to 1. There were no changes in terms of cultural aspects or language used in the six main parts of the MHQ. However, the authors valued an expert's suggestion for the section on patient characteristics in the MHQ-Thai version. The following revisions have been made in the Thai version questionnaire: (1) the item “ethnic background” was removed, (2) the item “changing jobs after having a hand injury” was modified to “clarify jobs before and after the injury” with the choice of “no job” added, (3) the range of income choice in the item “income” was changed, (4) the item “family income” was added, and (5) the item “medical expense reimbursement plan” was added.

#### 4.1.2. Testing of the Prefinal Version

Ten patients with orthopedic hand injuries, of whom eight were female and two were male, participated in the field test. All of them were right handed. Eight patients had right hand injuries while the rest had left hand injuries. All the patients could answer the MHQ-Thai version without any assistance from the researcher. They had no difficulty in understanding the questionnaire. Therefore, none of them recommended any changes in the language used. The final version was approved and will become available on the MHQ website (http://mhq.lab.medicine.umich.edu/).

### 4.2. Psychometric Testing of the MHQ-Thai Version

The demographic data for the 30 patients with hand injuries are shown in Table 1. There were nine males and 21 females with an age range of 29-74 years (average age = 51.86, SD ± 11.13 years). Most of them were right handed. Most participants had a hand and arm injury on the one side of the body, except for three patients who had hand injuries on both sides. The common causes of injury were bone fractures and muscle and soft tissue injuries. Fifty percent of the patients, who had worked before the injury, could not return to work after it.

The MHQ scores for the six domains are shown in Table 2. As a result of their injuries, the patients' lives were impacted in all six domains. The average total scores for the MHQ-Thai version for the first and second visits were 64.79 (SD ± 18.75) and 67.18 (SD ± 19.58).

#### 4.2.1. Construct Validity

Each domain in the MHQ-Thai version was correlated with the other domains with different levels of correlation coefficients, ranging from low to high, as shown in Table 3. Interestingly, the satisfaction domain had a high correlation with the overall function (*r* = 0.807, *p* < .001), ADL (*r* = 0.743, *p* < 0.01), and pain domains (*r* = −0.798, *p* < 0.01) and had a moderate correlation with the work (*r* = 0.648, *p* < 0.01) and aesthetics domains (*r* = 0.507, *p* < 0.01). In addition, the pain domain had a high correlation with the ADL domain (*r* = −0.731, *p* < 0.01) and a moderate correlation with the overall function and work domains with correlation coefficients of *r* = −0.693 and *r* = −0.605, respectively (*p* < 0.01).

#### 4.2.2. Internal Consistency

Internal consistency was good for the MHQ-Thai version (*α* = 0.835) as evidenced in Table 4. Also, internal consistency was satisfactory for each domain, except for the aesthetics domain, where Cronbach's *α* ranged from 0.778 to 0.992. Regarding the aesthetics domain, Cronbach's *α* coefficient was slightly lower than 0.5, which indicated unacceptable internal consistency.

#### 4.2.3. Test-Retest Reliability

Table 5 shows the test-retest reliability of the questionnaire. The calculated ICC for the total score of the MHQ-Thai version was 0.953, indicating excellent reliability. In addition, the test-retest reliability of the overall function, pain, and satisfaction domains was excellent with an ICC value that was greater than 0.90. The remaining domains, including ADL, work performance, and aesthetics, demonstrated good test-retest reliability, with the ICC values ranging from 0.776 to 0.882.

## 5. Discussion

This study was aimed at translating the MHQ into Thai and validating the MHQ-Thai version. The study showed that the MHQ was translated into Thai without any major changes. The MHQ-Thai version obtained good validity and reliability results when tested.

To achieve linguistic and cultural equivalence between the original version and the translated version of the questionnaire, its translation and adaptation required the use of a unique method [7]. Therefore, Beaton's protocol for cross-cultural adaptation was adopted in the translation process [7]. No major issues were observed during the translation process.

The content validity of the MHQ-Thai version was satisfactory. A slight adaptation was made on the demographic data collected regarding patient characteristics. To suit the Thai context, ethnic background, job, income, and medical expense reimbursement plan were adjusted. The item on the ethnic background was taken out because the population of Thailand is relatively homogeneous [13], and the vast majority of the population is Thai [14]. Also, the MHQ-Thai version will be used for patients who read and speak Thai. Thus, the ethnic background was considered unnecessary. The choices in the item on income were changed based on the average monthly income in Thailand [15]. An item on family income was added to the MHQ-Thai version to reflect the demographic characteristics of the extended family in Thailand. Lastly, an item was added to the MHQ-Thai version to include a question on any medical expense reimbursement plan as it was considered likely to have an impact on continuity of treatment.

During the testing phase of the prefinal version of the MHQ-Thai version, the respondents had no difficulty in completing the questionnaire. Therefore, the results of this study indicate that the MHQ was successfully cross-culturally adapted into Thai, which was similar to conclusions reached when it had been translated into other languages [6]. The construct validity of the MHQ-Thai version was also examined. Overall function, ADL, pain, work, and aesthetics were significant variables in predicting the satisfaction of Thai patients with hand injuries. Patients who had less pain score and high aesthetics and function scores would be expected to have high satisfaction score. In addition, pain was a good predictor of hand function, including overall function, ADL, and work. Patients who reported high pain scores would be expected to endorse difficulty with functioning, ADL, and work performance. In contrast, aesthetics had a low-to-moderate correlation with hand function, even though it was an important outcome for patients with a hand injury. The construct validity of the MHQ-Thai version was in line with the original MHQ and other translations [5, 16].

An instrument achieves reliability if it produces consistent, reproducible results on repeated administration [17]. The MHQ-Thai version revealed high internal consistency, which was in line with the original MHQ and the MHQs in other languages [5, 16, 18, 19]. Various translations still retained similarly high Cronbach's alpha coefficient even after cross-cultural adaptation. Each domain also had good-to-excellent internal consistency, except for the aesthetics domain. The unacceptable internal consistency of aesthetics found in this study was different from the original MHQ [5]. The low internal consistency of the aesthetics domain might be a result of poor interrelatedness between the four items in the aesthetics domain for each hand [20].

The ICC of the total score of the MHQ-Thai version showed excellent test-retest reliability for patients with hand injuries. The ICC for each domain was also good to excellent, which was a similar result to the original MHQ and the other translated MHQs [5, 16, 18, 19, 21, 22]. This indicated that the domains in the MHQ-Thai version were highly reliable in repeated testing and were stable over time.

Even though the MHQ-Thai version was a valid and reliable questionnaire, some limitations were found in this study. Beaton et al. suggested that 30 to 40 participants were required to test the prefinal version of the translation [7]. In this study, only ten participants with hand injuries took part. However, their understanding and interpretation of the questionnaire were carefully investigated. Another limitation was the relatively small number of subjects in the present study, which limits the ability to perform exploratory factor analysis (EFA). However, this may not affect the validity of the findings in the present study because the tested questionnaire was translated from a well-validated instrument and hence it is not essential to conduct EFA on this questionnaire. Despite the fact that the minimum sample size of 30 may provide relatively low statistical power of 0.51 [8], such power was calculated based on correlation or causation analysis. This study was, on the other hand, designed to test the validity and reliability of the questionnaire. Although there is no definite guideline to determine the appropriate number of sample size for questionnaire development, larger samples are always better than smaller samples. Hence, future studies should employ a larger number of sample size with an appropriate sampling technique to ensure a variety of participants with other types of hand injuries which is required to extend knowledge. Moreover, this study did not record the time taken to complete the MHQ-Thai version. Having information on average time taken to complete the questionnaire would be useful for health professionals. It would help them in deciding whether, in busy therapeutic settings, it is feasible for their patients to complete the outcome assessment.

## 6. Conclusions

The MHQ was translated and cross-culturally adapted into Thai. The MHQ-Thai version performed well in both validity testing and reliability testing, which was similar to the original MHQ and other translated versions. As long as the questionnaire is reliable and valid, the MHQ-Thai version could be used as an outcome measurement for self-perception of Thai patients with hand injuries.

## Figures and Tables

**Table 1:** Demographic data of participants (*n* = 30).

Demographic data	Number of patients (%)
Gender	
Male	9 (30)
Female	21 (70)
Education	
None	1 (3.33)
Primary education	8 (26.67)
Secondary education	9 (30)
Diploma	7 (23.33)
Undergraduate	5 (16.67)
Graduate	0 (0)
Dominant side	
Right	25 (83.33)
Left	5 (16.67)
Affected side	
Right	15 (50)
Left	12 (40)
Both	3 (10)
Cause of injury	
Bone injury: fracture	14 (46.67)
Joint degeneration: arthritis and subluxation	3 (10)
Muscle, tendon, and ligament injury	11 (36.67)
Nerve injury	1 (3.33)
Unknown	1 (3.33)
Previous work	
Yes	24 (80)
No	6 (20)
Occupation after injury	
Yes	12 (40)
No	18 (60)
Income	
Up to 5000 baht	9 (30)
5000-10,000 baht	9 (30)
10,001-15,000 baht	4 (13.33)
15,001-20,000 baht	4 (13.33)
Above 20,000 baht	4 (13.33)
Family income	
Up to 10,000 baht	4 (13.33)
10,001-20,000 baht	7 (23.33)
20,001-30,000 baht	10 (33.33)
30,001-40,000 baht	6 (20)
Above 40,000 baht	3 (30)
Medical expense reimbursement plan	
Yes	25 (83.33)
Social security scheme	6 (20)
Universal coverage scheme	11 (36.67)
Government enterprise officer	8 (26.67)
No (self-supporting)	5 (16.67)
Worker compensation scheme	
Yes	6 (20)
No	24 (80)

**Table 2:** The MHQ-Thai version score for the six domains (*n* = 30).

Domains	First assessment		Second assessment	
	Mean	SD	Mean	SD
Overall function	65.50	18.88	68.92	18.66
ADL	62.18	23.87	65.89	26.99
Work performance	46.17	27.09	45.17	27.50
Pain	23.83	21.99	21.75	21.25
Aesthetics	72.09	15.13	75.21	16.41
Satisfaction	66.67	23.40	69.65	23.16
Total	64.79	18.75	67.18	19.58

**Table 3:** Construct validity of the MHQ-Thai version.

	Spearman's rho	Overall function	ADL	Work performance	Pain	Aesthetics
Overall function	*r* Sig					
ADL	*r* Sig	0.671^∗∗^ 0.000				
Work performance	*r* Sig	0.369^∗^ 0.045	0.706^∗∗^ 0.000			
Pain	*r* Sig	−0.693^∗∗^ 0.000	−0.731^∗∗^ 0.000	−0.605^∗∗^ 0.000		
Aesthetics	*r* Sig	0.403^∗^ 0.027	0.694^∗∗^ 0.000	0.428^∗^ 0.018	−0.355 0.055	
Satisfaction	*r* Sig	0.807^∗∗^ 0.000	0.743^∗∗^ 0.000	0.648^∗∗^ 0.000	−0.798^∗∗^ 0.000	0.507^∗∗^ 0.004

Notes: ^∗^correlation was significant at the 0.05 level (2-tailed). ^∗∗^Correlation was significant at the 0.01 level (2-tailed).

**Table 4:** Internal consistency of the MHQ-Thai version.

Domains	Cronbach's *α* coefficient
Overall function	0.778
ADL	0.992
Work performance	0.892
Pain	0.947
Aesthetics	0.477
Satisfaction	0.850
Total	0.835

**Table 5:** Test-retest reliability of the MHQ-Thai version.

Domains	ICC	95% confidence Interval		*F* test with true value 0			
		Lower bound	Upper bound	Value	df1	df2	Sig
Overall function	0.912	0.824	0.957	21.791	29	29	0.000
ADL	0.882	0.767	0.942	15.928	29	29	0.000
Work performance	0.776	0.581	0.887	7.937	29	29	0.000
Pain	0.941	0.881	0.972	33.133	29	29	0.000
Aesthetics	0.799	0.619	0.899	8.933	29	29	0.000
Satisfaction	0.963	0.924	0.982	53.190	29	29	0.000
Total	0.953	0.904	0.977	41.534	29	29	0.000

Note: ICC (3,1) absolute agreement.

## Data Availability

The data used to support the findings of this study are available from the corresponding author upon request.
